# Optimizing clinical documentation in virtual hospitals: a task-technology fit perspective

**DOI:** 10.1093/jamiaopen/ooag095

**Published:** 2026-06-12

**Authors:** Adeola Bamgboje-Ayodele, Adrian Boscolo, Melinda Dao, Jessica Yi, Julie Li, Miranda Shaw, Owen Hutchings, Steven McPhail, Melissa Baysari

**Affiliations:** Discipline of Design, School of Architecture, Design and Planning, The University of Sydney, Australia; Sydney Local Health District, Australia; Sydney Local Health District, Australia; Digital Health Human Factors Research Group, Sydney Nursing School, Faculty of Medicine and Health, The University of Sydney, Australia; Digital Health Human Factors Research Group, Sydney Nursing School, Faculty of Medicine and Health, The University of Sydney, Australia; Sydney Local Health District, Australia; Sydney Local Health District, Australia; Australian Centre for Health Services Innovation and Centre for Healthcare Transformation, Queensland University of Technology, Brisbane, 4059, Australia; Digital Health Human Factors Research Group, Sydney Nursing School, Faculty of Medicine and Health, The University of Sydney, Australia

**Keywords:** virtual hospital, clinical documentation, task re-design, process, AI scribes

## Abstract

**Objective:**

Virtual hospitals differ from brick-and-mortar hospitals across many elements of the socio-technical work system, particularly their complete reliance on digital tools for communication and care coordination. It is currently unclear whether existing initiatives to improve clinical documentation in brick-and-mortar hospitals are fit for purpose in virtual hospital settings. This study aimed to examine the documentation experiences of clinicians when using technologies to support care delivery in a virtual hospital and to identify fit-for-purpose initiatives to optimize documentation processes.

**Materials and Methods:**

We performed focus groups and a co-design workshop with 28 nursing and medical staff at a virtual hospital. Focus group data were first analyzed inductively, and later deductively mapped to the task-technology fit framework. Field notes from the workshop were recorded, digitized and analyzed thematically.

**Results:**

We identified the unique characteristics of tasks and technologies used in a virtual hospital and found problems with data findability and difficulty in using multiple technologies concurrently, arising from the technology-mediated nature of virtual care, which led to performance impacts on clinicians (eg, inefficiency, duplication of work) and potential impacts on patients (limits time for patient care). Also, we identified opportunities where technologies could be used to streamline documentation (eg, automatic generation of handover notes, reduction of free-text entries, use of AI scribes), opportunities for streamlining documentation tasks (ie, matching documentation requirement to nature of encounter), and strategies for optimizing the escalation process in virtual hospitals (ie, removing extra escalation layers and aligning more closely with escalation practices in brick-and-mortar hospitals).

**Conclusion:**

Whilst tasks and technologies used for care delivery differ between virtual and traditional hospitals, documentation problems and impacts are fairly consistent across settings. However, the virtual environment amplifies these challenges due to clinicians’ dependence on multiple digital systems. We identified a clear need for functionality that supports a team-based approach to virtual care delivery and a need for policy review, escalation process review and clinical task re-design prior to incorporating additional technologies to minimize documentation burden (eg, AI scribes) in virtual settings.

## Background

Comprehensive and accurate clinical documentation is essential for patient safety and high quality healthcare delivery.^1^ Clinical documentation can facilitate clinician sense-making and synthesis of a patient’s case, while enabling shared understanding in clinical teams.^2^^,^^3^ However, evidence suggests that the impacts of electronic documentation have been mixed.^4^ For example, some studies have identified unintended consequences of electronic documentation such as inefficiency, clinician burden, dissatisfaction and burnout.^5–10^ Various improvement initiatives and strategies have been employed to reduce clinician burden and improve clinician experience of electronic documentation such as audit/feedback, dictation, templates, and reminders.^1^^,^^11^ A systematic review of 11 systematic reviews on quality criteria and requirements for nursing documentation, identified a key improvement strategy to be aligning documentation with nursing processes.^12^ However, previous empirical studies and reviews have focused on clinician documentation processes in brick-and-mortar hospitals and limited work has examined documentation processes and needs in virtual hospital settings.

Virtual hospitals are models of care in which hospital‑linked services are delivered remotely and supported entirely by digital technologies, where clinicians and patients are not physically co-located.^13^^,^^14^ Virtual hospitals are relatively new but are increasingly being adopted internationally.^15^ Existing evidence suggests that virtual hospitals differ from brick-and-mortar hospitals across many elements of the socio-technical work system: physical environment, people, tools and technologies, organizations and processes, highlighting fundamental workflow differences.^16^ Recent reviews highlight substantial variability in virtual hospital models and note that research has largely focused on remote monitoring and care delivery structures rather than documentation workflows.^17^ To date, no studies have examined clinical documentation practices in virtual hospital settings or applied a task–technology fit perspective to this context.

Our prior work system analysis revealed duplication of work to be a barrier to clinicians documenting vital signs and symptoms in a virtual context. Clinicians in that study reported the need to manually enter notes and data including vital signs and symptoms, across multiple disconnected systems (eg, EHR, dashboard notes).^18^ This study represents the next phase of work, where we sought to understand in-depth the documentation work in this setting. Questions remain as to whether existing clinical documentation improvement initiatives (eg, templates) in brick-and-mortar hospitals are fit for purpose in virtual hospital settings. The aim of this study was to explore the documentation experiences of clinicians when using technologies to support care delivery in a virtual hospital and to identify potential solutions to optimize documentation processes. To our knowledge, this is the first study to investigate clinical documentation practices in a virtual hospital setting using task‑technology fit as a conceptual model.

## Materials and methods

### Design

We used experience-based co-design (EBCD) to understand clinician documentation experiences. EBCD methods focus on experiences as a whole,^19^ and can combine one or more approaches (eg, workshops, interviews, focus groups) of engaging with clinicians.^20^^,^^21^ We drew on elements of experience-based co-design (EBCD) to guide our engagement with clinicians, including facilitated focus groups, participatory workshops, and collaborative identification of improvement opportunities. Although traditional EBCD typically involves both patients and staff, our study focused specifically on clinician documentation practices, tasks performed exclusively by clinicians. For this reason, clinicians were the stakeholder group engaged for this adapted EBCD approach. We applied EBCD’s co-design principles to generate shared understanding of current workflows and collaboratively identify solutions relevant to documentation in the virtual hospital setting. Using EBCD methods, we conducted a two-phased qualitative study and have used the COREQ checklist to report our findings (Supplementary Appendix A).

### Setting

Our study was conducted at a virtual hospital in Australia. Details on the setting have been described previously.^18^ In summary, the virtual hospital primarily caters to adult patients and is staffed by a multidisciplinary clinical team which comprised of 20 doctors and 37 nurses at the time the study was conducted. The virtual hospital uses care pods, remote monitoring tools, and a clinical dashboard to track patient data–including vital signs and demographics–via both automated and manual data entry pathways.

### Participants and procedure

#### Phase 1: Focus groups

To capture interprofessional dependencies in documentation, we purposefully included both nurses and doctors and conducted two profession‑specific focus groups. All nursing and medical staff at the virtual hospital were eligible to participate in a focus group. Using a convenience sampling approach, a clinician-researcher on our research team invited all eligible participants to take part in a focus group via email. Following this, clinicians directly emailed the researcher to volunteer to participate in the study. A postdoctoral researcher (ABA) experienced in conducting qualitative research, explained the purpose of the study, and facilitated the online sessions. During the focus groups, findings from our prior work system analysis were presented.^18^ Participants were then asked to answer six questions, as shown in Table 1. Data were collected in June 2024. The focus group discussions were video recorded, and transcribed.

**Table 1.** Focus group question guide for Phase 1.

Can you please describe the main things you document before, during and after your encounter with a patient, including the preparation of handover documents?
Where, and in what systems, are these tasks documented?
How does using a number of different technology platforms affect your documentation process?
Of those things you mentioned previously, what documentation tasks do you think are unnecessary?
Is there any double documentation? ie, where you have to record the same thing twice (or more than twice)?
What are your thoughts on how task duplication can be minimised?

Although the focus group questions did not explicitly ask clinicians to compare virtual and brick‑and‑mortar documentation practices, participants frequently drew on their experience in both settings, enabling these comparisons to emerge naturally during inductive analysis.

#### Phase 2: Workshop

All nursing and medical staff at the facility, and information technology (IT) staff and vendor representatives involved with the design and implementation of virtual care technologies at the site, were eligible to participate in a workshop. Using a convenience sampling approach, the clinician-researcher on the team championed the project within the health service and invited all eligible participants via email to the workshop. A postdoctoral researcher (ABA) experienced in conducting qualitative research, explained the purpose of the study, and facilitated the sessions together with either MB, MD, JY, or JL. The in-person sessions occurred at the University of Sydney. Participants were then divided into small groups of 4-6 people, to facilitate discussions, and were asked to answer two questions: (1) focusing on what you document (ie, your tasks), how can we streamline documentation for nurses and doctors? and (2) focusing on where you document (ie, the technologies), how can we streamline documentation for nurses and doctors? Data were collected in August 2024. Field notes were handwritten and digitized following the workshop.

### Data analysis

Both phases produced sufficiently rich accounts of clinicians’ documentation practices, with participants offering detailed examples that supported robust thematic development. Practical constraints, including clinician availability and the need to minimize disruption to patient care informed the decision to stop data collection. Interviews were analyzed using both an inductive and deductive thematic analysis approach.^22^ Our analytical approach included data familiarization, coding, generating initial themes, reviewing potential themes, defining and naming themes, and producing the report.^22^ For Phase 1, inductive thematic analysis was conducted by an experienced researcher with expertise in human factors, virtual care and health technology evaluation (ABA) and reviewed by a second researcher, a junior researcher with expertise in health services research (JY). A third researcher (MB), a professor with expertise in human factors and health technology evaluation, reviewed all transcripts and coding for accuracy and consistency. Two researchers (ABA and MB) met frequently throughout data collection and analysis to discuss emerging themes. Disagreement in themes were discussed until a consensus was reached.

Following this, we conducted deductive thematic analysis as themes were later mapped to the task technology fit (TTF) model. The TTF model provides a lens to understand how technology is used and the value it creates.^23^ The TTF model asserts that the alignment of the task requirements and the technology characteristics that enable a user to perform the tasks results in performance.^24^^,^^25^ TTF is the extent to which a technology assists a person to perform their tasks and this is shaped by the interaction between task characteristics and technology functionalities.^23^^,^^26^ When measuring fit, dimensions considered are data quality, data locatability, authorization to access data, data compatibility, ease of use/training, production timeliness, systems reliability, and information system relationship with users.^23^

For Phase 2, data from the field notes underwent inductive thematic analysis. To ensure scientific rigor, member checking was conducted as emerging themes were presented to some participants and key stakeholders, and feedback received informed final refinement of the themes.

### Ethical considerations

Ethics approval was obtained from the hospital’s Human Research Ethics Committee (X21-0362 & 2021/ETH11708). Consent was obtained from all participants, who were able to withdraw from the study at any time.

## Results

### Phase 1: Focus group results

A total of 15 participants took part in two focus group sessions which lasted 52 minutes on average. The demographic characteristics of participants are shown in Table 2.

**Table 2.** Demographic characteristics of participants in Phase 1.

Demographic characteristic	*n* (%)
Role	
Nurse	11 (73)
Doctor	4 (27)
Time in role	
0-1 year	2 (13)
1-2 years	2 (13)
2-5 years	5 (33)
More than 5 years	1 (8)
Not reported	5 (33)
Years of work experience	
0-4 years	2 (13)
5-9 years	3 (20)
10-14 years	3 (20)
15-19 years	0 (0)
20 years and above	2 (13)
Not reported	5 (33)
Sex or sex assigned at birth	
Male	3 (20)
Female	12 (80)
Age (years)^a^	
Mean	38
Range	26-64

a Missing data (*n* = 5).

**Figure 1.**
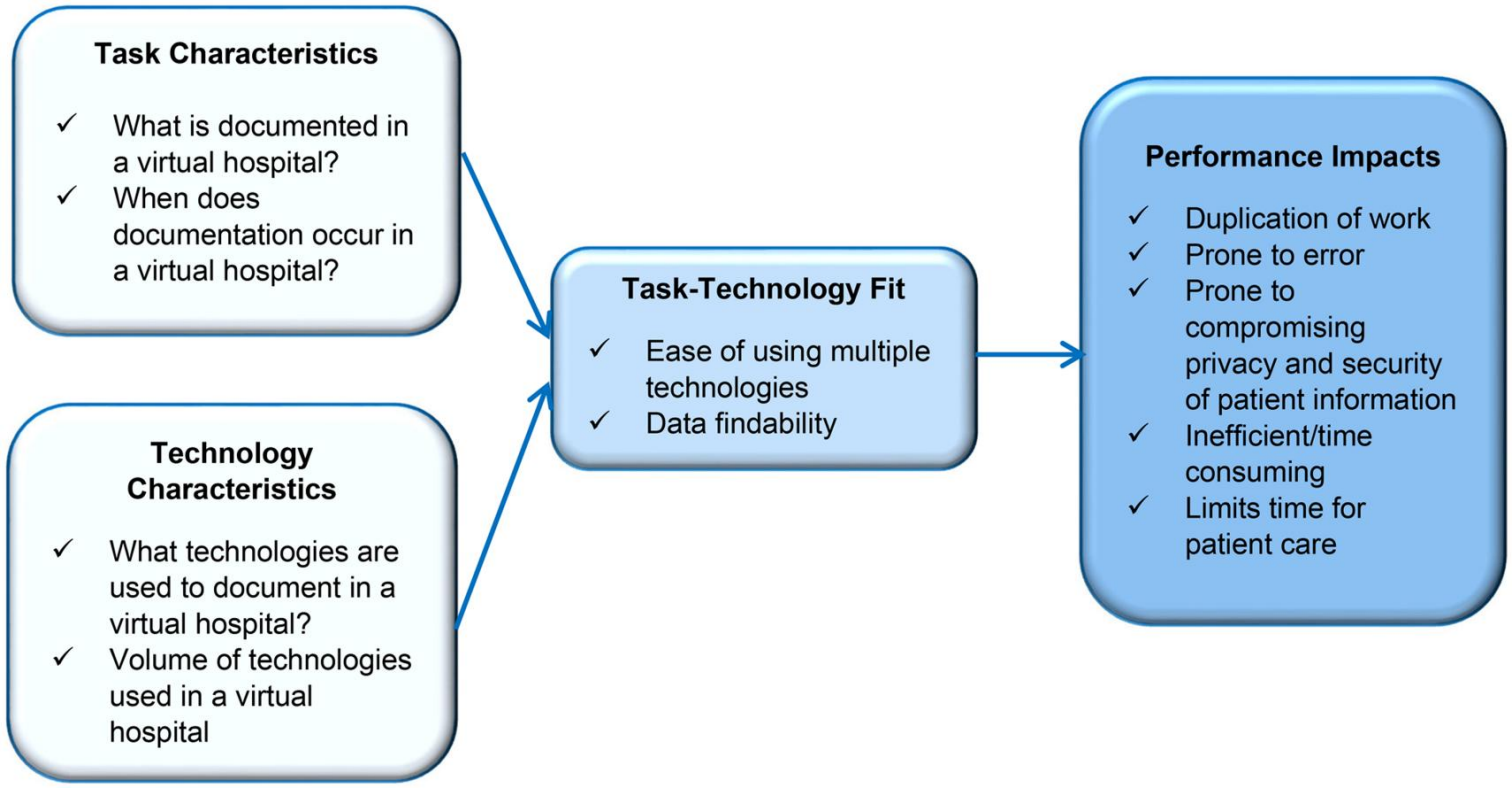
Themes mapped to task technology fit framework.

Our findings revealed 11 sub-themes that were mapped to the domains of the task technology fit model. Of these, two were mapped to task characteristics, two to technology characteristics, two to task-technology fit, and five to performance impacts. See Figure 1.

## Task characteristics

Tasks reported by participants in this study focused on conducting patient reviews, case conferences, intake meetings and handover. What and when documentation occurred are sub-themes listed below.

### What is documented in a virtual hospital?

Participants reported that a range of clinical and administrative information relevant to care delivery were typically recorded in the virtual hospital. Clinical information comprised clinical assessments, treatment plans for each patient, status of patient reviews, and handover notes: “[we document] *our assessment and whatever the outcome of the assessment is, the plan for that*” – P5, nurse. More administrative-type information included patient details, patient education and activity-based funding: “*so on EMR, it’s usually everything. So patient details, clinical review notes, observations, plans, everything*.” – P1, doctor.

### When does documentation occur in a virtual hospital?

Participants explained that when documentation occurred depended on the task being performed. For example, participants reported documenting before, during and after case conferences and intake meetings: “*So we document on a call log that’s based in teams when the call comes in. And then we usually send an email to the referrer … for the referral form and then that gets uploaded to EMR, and then we have a MDT [*multidisciplinary team*] meeting, and that’s also documented in EMR, and there’s also a spreadsheet in teams where we document the outcomes there, and [the relevant clinician] actions it, and… there’s email follow up regarding that too, or phone calls, and … there’s no patient contact in that whole journey.”* – P1, doctor. However, documentation occurred only during and after patient reviews, not before. Participants also reported that they documented at the beginning and end of shift handover and per patient handover: “*In terms of handover, … that needs to be updated at least the evening before, and then kind of reviewed the morning of as well. And then, once the handover is complete*” – P2, doctor.

## Technology characteristics

### What technologies are used for documentation in a virtual hospital?

A total of 10 technologies were reported to be used for documentation tasks in the virtual hospital. Four technologies were used primarily for communication (ie, Outlook, Microsoft Teams’ chat function, SMS broadcast app, Genesis), four to support notetaking (ie, Microsoft Word, Excel, Notepad, remote desktop), one for logistics (ie, Miya dashboard), and the other for documenting patient data (ie, the EMR).

### Volume of technologies used in a virtual hospital

Participants reported that they used too many mediums of communication technologies and this was “*overwhelming*” – P1, doctor. It was specifically stated that each technology was an additional tool they needed to keep track off: “*and then on top of that, we’ve also got to manage our email inbox, which is where a lot of the communications is happening*” – P12, nurse.

Despite the use of multiple technologies, participants explained that many of their processes were manual, not automated. There was a view that some of the technologies were not being used to their full potential. For example, a participant said: “*a lot of the processes with us is not very high tech. So a lot of the things we’re doing are manual…we’re probably not utilizing as much what Miya can do like you know, maybe they can pull out those reports out on EMR, but a lot of things we’re having to record manually*” – P5, nurse.

The use of many technologies without single-sign-on technology was seen to be challenging in the virtual care setting. Participants reported that manually logging in to multiple platforms was time consuming, which was exacerbated by the time-out functionality of many of the technologies: “*When you come to work in the morning, probably it will take 15 minutes just to log into everything and get ready. Because there’s so many of them you have to open*.” – P13_nurse. Another participant said: “*they all like to log themselves out every so often, and they all like to do it at different times. So you’re not just logging in once a day. You might be logging in 10 times a day when everything’s timed out at various times, which just makes logistically, it’s just hard*” – P2, doctor.

## Task-technology fit

It was reported that the many technologies used for clinical documentation in the virtual hospital setting made information hard to find, and were not easy to use, when viewed as a whole, which led to workarounds.

### Ease of using multiple technologies

The use of multiple technologies to support documentation reportedly led to clinicians using additional software, which further increased the number of technologies in the work environment: “*And then a lot of us … have like a Microsoft document or an Excel sheet or something just for all our different documentations that we can then copy and paste in those various platforms*.” – P12, nurse. Another participant stated that: “*I also write on the [electronic] notepad beforehand, so I don’t lose it, or if I need to copy it into multiple places, that’s sort of my go to*” – P3, doctor.

Participants also reported using additional hardware and paper to support their use of multiple technologies for documentation. They noted that the sheer volume of technologies required them to use additional monitors (or screens): “*I had to buy another monitor at home. And sometimes I use my laptop, so that’s a third screen*.” – P1, doctor. In addition, another participant explained that the use of physical notebooks was a necessary workaround to support her documentation task: “*I only work two days a week but half a book is 6 months worth of work so, you know, this is an A5. I fill a few of these each day as I’m talking to people, take notes before we all take notes before we write*” – P9, nurse.

### Data findability

With multiple technologies being used, participants reported that this made information hard to find and retrieve: “*it’s hard to find exactly what you need, because it’s tucked away in a folder somewhere that you may not have access to*” – P2, doctor.

## Performance impacts

The task characteristics, technology characteristics, and the fit between them reportedly impacted clinician performance. Participants noted that duplication of work was prone to error. They also noted the potential to compromise the privacy and security of patient information. All identified impacts reportedly resulted in inefficiencies which limited the time for direct patient care.

### Duplication of documentation

Participants reported that duplication of documentation occurred during various tasks including during handover, patient review, patient management, meetings and referral processes for some care models. Box 1 presents an example as to how duplication reportedly occurred during patient reviews.

Box 1. Duplication of documentation during patient reviews

The outcome of the nursing assessment is documented by email which is typically sent to the nurse unit manager (NUM) if the patient’s case needs to be escalated for a review. The NUM then forwards the nurse’s email to the medical officer for review. In addition, the nurse documents the purpose of the patient review on a clinical information system (not the EMR), and the NUM uses that information to triage the patient, assigning them to a doctor for review. “*we’ll document our assessment and whatever the outcome of the assessment is, the plan, … if we do need to escalate for review, then we’ll email the NUM and that will be forwarded to the MO (medical officer). So that’s two documentation and then actually updating Miya on what the review is for and the NUM will use Miya as a booking platform for triage and for booking for the doctor’s review” –* P5, nurse.

A participant noted that the same documentation was sometimes required to be completed in three places: “*So we have a cohort for … palliative care calls and after we’ve done the assessment, we need to document that in EMR but then we’ve also got additional call logs which are spreadsheets …[where we] document the same thing in. … So you put the same information into an email to send to the nursing manager as well as to another external team. So that’s three places now where that same information is*” – P12, nurse.

Also, participants described a new handover process that required documenting the same information in multiple places: “*like this new handover process, you’re updating a Word document and then you’re writing …another kind of clinical note into EMR. So … I think the double doing of it [documentation] is a bit cumbersome*” – P2, doctor.

In addition, during referral processes which typically involve clinician-to-clinician meetings, duplication of work was seen as a problem: “*So we document on a call log that’s based in teams when the call comes in. And then we usually send an email to the referrer … for the referral form, and then that gets uploaded to EMR, and then we have a MDT meeting, and that’s also documented in EMR, and there’s also a spreadsheet in teams where we document the outcomes there…and then it gets actioned, and … there’s email follow up regarding that too, That’s just purely documentation about referral processes*” – P1, doctor.

### Prone to error

Duplicating information across multiple technologies was viewed to be prone to error as the clinician could forget to update the information in all the required places: “*Sometimes, I think, Oh my God, I forgot to put it in one place. Like I’ve taken a phone call, I’ve documented it in the EMR, I’ve told the NUM, and then I think, Oh my God, there’s a spreadsheet I’ve got to add to as well that I took a phone call.*” – P11, nurse. In addition, it was noted that clinicians could potentially type the wrong information: “*it can also increase human error because if any of the wording is slightly different and it’s not the same as what you’ve documented in EMR, then that can cause a problem because now it’s misinformation”* – P12, nurse. When further probed about whether participants could use the copy and paste functionality when duplicating information across multiple platforms, they noted that it was not as straightforward due to the need to summarize the information: “*We can* [copy and paste], *but what you document in EMR is quite a lot for what you can put in the spreadsheets because it is meant to be a summary. And so you still do need to edit and … it’s always free text, so there’s always that problem of misspelling, miswriting, missing information that can happen when you’re having to put information in different places*” – P12, nurse.

### Potential to compromise privacy and security of patient information

Participants reported that using physical notebooks and paper to support clinical documentation posed a risk to the privacy and security of patient information: “*I have a paper notepad also. Okay. More to do list. Do not forget, there’s patient details on this* [paper notepad]” – P1, doctor.

### Inefficiency

Documentation was viewed to be time consuming and participants explained that the process of documentation in various technologies could take longer than the actual virtual consultation. For example, a participant noted that, “*the discussion itself could be only 15 minutes, but then you’re spending another half an hour doing documentation. So that’s double the amount of time.*” – P12, nurse. Participants discussed that typing during the consultation could save time, but this was viewed as potentially undermining the clinician-patient relationship: “*we know that sometimes, it’s more time efficient to be typing while we’re talking about. Sometimes you just want to be in the moment and just make yourself like you’re present for your patient, that you’re not someone there just to document what they’re saying, but you’re actually listening to them*.” – P5, nurse. Participants also discussed emerging technologies, such as AI‑enabled scribe tools, as a potential way to reduce the tension between documentation and maintaining presence during virtual consultations whilst minimizing inefficiency. For example, one nurse described: “*a group of doctors have developed i-scribe which uses AI so that you don’t have to be typing away while you’re interviewing a patient. So you’re talking directly to the patient and you’re in the moment, you’re present… So I think AI will be helpful for us*.” – P9, nurse.

### Reduces time for patient care

A key concern raised by participants was the potential impact of documentation on patient care. Participants expressed frustration as documentation was negatively impacting the time they could dedicate to direct patient care. For example, a participant stated that, “*the frustration is that you’re just doing all this documentation all day instead of seeing patients. … you’ve got 10 reviews waiting for respiratory and each review takes an hour, but it doesn’t take an hour because it takes you an hour to sit with the patient. It takes an hour because you’ve got all this kind of stuff in the background that needs to get done*” – P2, doctor.

### Phase 2: Workshop results

A total of 13 participants took part in the documentation optimization workshop which lasted 75 minutes. These participants were different from those who took part in Phase 1. The demographic characteristics of participants are shown in Table 3.

**Table 3.** Demographic characteristics of participants in Phase 2.

Demographic characteristic	*n* (%)
Organisation	
Virtual hospital	8 (53)
District IT staff	2 (13)
Vendor	3 (20)
Role	
Nurse	5 (33)
Doctor	3 (20)
Non-clinical	5 (33)
Time in role	
0-1 year	5 (33)
1-2 years	2 (13)
2-5 years	4 (27)
More than 5 years	2 (13)
Years of work experience	
0-4 years	1 (7)
5-9 years	4 (27)
10-14 years	3 (20
15-19 years	1 (7)
20 years and above	4 (27)
Sex or sex assigned at birth	
Male	3 (47)
Female	10 (53)
Age (years)	
Mean	41
Range	30-57

Abbreviations: NUM, nurse unit manager; IT, information technology.

The workshop outcomes were grouped into three themes: streamlining documentation tasks, streamlining escalation process, and using technologies to streamline documentation.

## Streamlining documentation tasks

To streamline documentation tasks associated with patient reviews, participants suggested that documentation should suit the nature of the encounter. For instance, documentation of an initial assessment should be detailed and comprehensive. However, templates for documentation of follow-up encounters could be streamlined, focusing on updates rather than a complete assessment.

## Streamlining escalation process

Nurses highlighted that a current patient review escalation pathway that was based on disaster response protocols introduced during the pandemic (see Box 1), was no longer relevant and required streamlining. Doctors reported that contact from the NUMs could occur via email, mobile, Microsoft Teams, or via all three means. To align more closely with escalation practices in brick-and-mortar hospitals, nurses recommended bypassing the NUM and directly contacting doctors via the clinical information system (eg, Miya), where details of the escalation are currently required to be documented, thereby simplifying the process and communication pathways.

## Using technologies to streamline documentation

Participants reported that there was a requirement for additional clinical documentation in a virtual hospital when compared to brick-and-mortar hospitals, stemming from the Medical Board of Australia’s telehealth guidelines. To minimize documentation, participants suggested automating some aspects of documentation and reducing free-text entries to satisfy but simplify compliance to the telehealth guidelines. Specifically, participants indicated a need for:

- Automatic generation of handover notes (currently recorded in Excel) from existing documentation in EMR and/or from other clinical information systems (CIS).
- Automatic population of medical review and logistical notes (from other CIS) into the EMR (eg, medical review templates).
- Ability to initiate a phone call through an existing CIS (eg, Miya Precision), with automatic documentation of the call, or via a tick-box function.
- Automatic generation of an SMS upon call scheduling in the CIS.
- A tick-box option in the CIS to indicate that a patient did not answer a call.

Other suggestions included the use of artificial intelligence (AI) scribes and the consolidation of all Microsoft Excel files into the one file with multiple worksheets for the different types of documentation.

Participants indicated that virtual care delivery practices should accommodate a team-based approach to care and reduce duplication of documentation. Specifically, they reported a need for a clinical note/summary that multiple clinicians could contribute to or cosign, to remove the need for every clinician who was present at a virtual encounter to create individual notes. Participants explained that the *Comprehensive Care Plan*, a single live document in the EMR that could be consistently updated by all clinicians involved in a patient’s care, is currently in use in brick-and-mortar outpatient settings and could potentially be customized for use at the virtual hospital.

## Discussion

In this study, we sought to examine the documentation experiences of clinicians when using technologies to support care delivery in a virtual hospital and to identify potential strategies to optimize documentation processes. We identified the unique characteristics of tasks and technologies used in a virtual hospital and highlighted that there are problems with data findability and difficulty in using multiple technologies concurrently which led to performance impacts on clinicians (eg, inefficiency, duplication of work) and potential impacts on patients (limits time for patient care). Also, we identified opportunities where technologies could be used to streamline documentation (eg, automatic generation of handover notes, reduction of free-text entries, use of AI scribes), opportunities for streamlining documentation tasks (ie, matching documentation requirement to nature of encounter), and strategies for optimizing the escalation process in virtual hospitals (ie, removing extra escalation layers and aligning more closely with escalation practices in brick-and-mortar hospitals).

Although the task and technology characteristics in virtual hospitals differ from that of brick-and-mortar hospitals, the documentation difficulties and impacts identified in our study are consistent with existing evidence on traditional hospitals. For example, a recent systematic review of 28 articles on issues contributing to documentation burdens in traditional clinical settings found usability concerns such as problems with data findability^27^. The review noted that fragmented information across multiple clinical information systems increases the time to locate and synthesize data, which contributes to documentation burden–aligning with our findings^28–30^. The impact of documentation burden on clinicians have been well-documented, with duplication of work^31^, inefficiency^32^, use of additional software as workarounds^33^, and increased risk of errors^27^ reported in existing studies conducted in traditional hospitals, echoing the results of our study.

While the technologies used in this virtual hospital are not inherently different from those used in traditional settings, the context of use is markedly different. We found that documentation issues (eg, duplication, data findability, multi‑system workarounds) are amplified in virtual settings because clinicians must rely solely on digital systems without the non‑digital pathways available in brick‑and‑mortar care. In virtual care, almost all clinical tasks including assessment, communication, escalation, and team coordination are fully technology‑mediated^18^. As a result, clinicians depend on a larger number of digital systems to replace functions that would otherwise occur in shared physical spaces (eg, hallway conversations, bedside handovers, ward rounds). These findings illustrate that the virtual context does not merely replicate documentation challenges seen in physical hospitals but intensifies them by increasing the number of technologies clinicians must navigate and by not providing alternative, non‑digital pathways for coordinating care.

COVID-19 drove the rapid adoption and implementation of virtual models of care including the establishment of virtual hospitals in many countries including Australia, the United States and the United Kingdom^34–36^. Globally, health systems have been transitioning into the new norm where virtual models of care are being expanded beyond respiratory conditions and are used to reduce pressure on traditional hospitals^37^^,^^38^. However, some policy requirements that upheld virtual models of care during the pandemic are not fit for purpose as evidenced by our study findings. We therefore call for a review of virtual care policies to better align with new ways of working, (ie, a team-based approach to care) and reduce clinical documentation burden.

In virtual hospital settings, evidence from this study and our previous works indicates that documentation is often duplicated not solely because of technological limitations, but because the underlying clinical tasks are fragmented or poorly defined^18^^,^^39^. When tasks are layered, overlapping, or inconsistently structured, documentation requirements tend to mirror this complexity resulting in inefficiencies and clinician frustration^27^. We found that, in some cases, documentation drives the task, while in others, the task dictates the documentation, creating a misalignment that hampers workflow. A more effective approach may be to first critically review and streamline the clinical tasks in virtual hospitals clarifying their purpose, sequence, and ownership, and then align documentation activities and technologies accordingly. Designing tasks first could help reduce duplication, improve clarity, and ensure that technology enhances rather than complicates care delivery.

As noted by participants in both the focus groups and the workshop, AI‑enabled scribe tools were identified as a potential strategy to reduce documentation burden. In recent years, there has been a rapid uptake of artificial intelligence (AI)-driven digital scribes that transcribe, summarize, and interpret clinical conversations to minimize clinical documentation burden^40^^,^^41^. Whilst promising, evidence of the effectiveness of AI scribes is limited, inconclusive and focused on brick-and-mortar clinical settings. A systematic review of eight articles evaluating the effectiveness of AI scribes in streamlining clinical documentation found that scribes may improve documentation time but there have been reports of poor documentation quality^42^. Another systematic review of 10 studies describing the effect of AI scribes on clinicians found that while documentation quality improved, inconsistencies in accuracy necessitated manual corrections and raised concerns about potential errors^43^. In virtual hospital settings, the impact of AI scribes on clinician documentation remains unclear. Given the complete reliance on technologies in virtual hospitals, introducing scribes adds to the technological ecosystem and warrants careful consideration to avoid amplifying existing complexities. Prior studies have called for adequate training and support, organizational preparedness, system interoperability, workflow integration and the critical consideration of the ethical dimensions of AI scribe implementation ^42^. Our study adds to this body of knowledge by shedding light on the importance of clinical task re-design to ensure that emerging technologies such as AI scribes are fit for purpose in virtual hospital settings.

### Strengths and limitations

This is the first study to investigate clinical documentation practices in a virtual hospital setting using task-technology fit as a conceptual model. Our two-phased qualitative research involved the purposive recruitment of 28 participants, ensuring all relevant groups are represented. Member checking strengthened our approach and ensured credibility and relevance of our findings to the stakeholder groups. A limitation of our study is its exploratory nature, focusing on a single virtual hospital, which may affect the transferability of the findings to other contexts and settings. Also, given the qualitative research design, the results are interpretive in nature and do not quantify the extent of the workflow implications identified. The relatively small sample, including 15 focus group participants, may constrain the breadth of perspectives captured. Participation was based on a convenience sample. As such, our findings may not fully reflect the diversity of clinician experiences and perspectives within the broader population, potentially shaping the nature and scope of the interpretations developed. Additionally, the disproportionate representation of nurses relative to doctors may limit the extent to which findings capture the distinct documentation practices of different professional groups. Taken together, these factors suggest that findings should be interpreted as contextually situated insights rather than comprehensive accounts of clinical documentation practices in virtual hospitals.

## Conclusion

In this study we explored in-depth the documentation experiences of clinicians when using technologies to support care delivery in a virtual hospital. Whilst tasks and technologies used for care delivery differ between virtual and traditional hospital settings, documentation problems and impacts are fairly consistent across settings. We identified a clear need for functionality that supports a team-based approach to virtual care delivery and a need for policy review, escalation process review and clinical task re-design prior to incorporating additional technologies to minimize documentation burden (eg, AI scribes) in virtual settings.

## Supplementary Material

ooag095_Supplementary_Data

## Data Availability

The datasets generated and analyzed during this study are not publicly available due to privacy or ethical restrictions.
